# Computational Methods in Psychotherapy: A Scoping Review

**DOI:** 10.3390/ijerph191912358

**Published:** 2022-09-28

**Authors:** Valeria Cioffi, Lucia Luciana Mosca, Enrico Moretto, Ottavio Ragozzino, Roberta Stanzione, Mario Bottone, Nelson Mauro Maldonato, Benedetta Muzii, Raffaele Sperandeo

**Affiliations:** 1 SiPGI–Postgraduate School of Integrated Gestalt Psychotherapy, 80058 Torre Annunziata, Italy; 2 Department of Neurosciences and Reproductive and Odontostomatological Sciences, University of Naples Federico II, 80131 Naples, Italy; 3 Department of Humanistic Studies, University of Naples Federico II, 80131 Naples, Italy

**Keywords:** psychotherapy, psychopathology, neural networks, patient–therapist relationship, complex systems, graph theory, machine learning

## Abstract

Background: The study of complex systems, such as the psychotherapeutic encounter, transcends the mechanistic and reductionist methods for describing linear processes and needs suitable approaches to describe probabilistic and scarcely predictable phenomena. Objective: The present study undertakes a scoping review of research on the computational methods in psychotherapy to gather new developments in this field and to better understand the phenomena occurring in psychotherapeutic interactions as well as in human interaction more generally. Design: Online databases were used to identify papers published 2011–2022, from which we selected 18 publications from different resources, selected according to criteria established in advance and described in the text. A flow chart and a summary table of the articles consulted have been created. Results: The majority of publications (44.4%) reported combined computational and experimental approaches, so we grouped the studies according to the types of computational methods used. All but one of the studies collected measured data. All the studies confirmed the usefulness of predictive and learning models in the study of complex variables such as those belonging to psychological, psychopathological and psychotherapeutic processes. Conclusions: Research on computational methods will benefit from a careful selection of reference methods and standards. Therefore, this review represents an attempt to systematise the empirical literature on the applications of computational methods in psychotherapy research in order to offer clinicians an overview of the usefulness of these methods and the possibilities of their use in the various fields of application, highlighting their clinical implications, and ultimately attempting to identify potential opportunities for further research.

## 1. Introduction

### 1.1. Background

The psychotherapeutic encounter, a specific organised and systematic relationship between a patient and a therapist, is a complex system to all effects [1].

The prevailing scientific models of the last century were founded on the idea of objectivity and a universe made up of isolated objects subjected to laws of linear causality [2,3,4]. In this context, explaining a phenomenon means discovering the simplest constitutive elements and the simple rules that build systems composed of the sum of the parts. For Morin [5,6], this hyper-simplification that makes us blind to the complexity of reality is a real pathology of reason.

In fact, since the beginning of the last century, it has become increasingly evident that facts of nature do not have the characteristics that make them accessible to classical scientific methods [2,4].

In the same way, psychic experiences, which are experienced essentially in the first person, often elude the possibility of being described in quantitative and third-person methods [7,8,9].

Nevertheless, the research programs of the psychic area, for almost the entire last century, have been oriented towards attempts to simplify the object of study.

Some approaches have refused to investigate the intrinsic processes of the mind, defining it as a “black box” [10]; others have roughly simplified the measurement of psychic experience.

Research programs in physics and biology, however, have addressed the issue of complexity and have produced epistemological reflections and methods more suited to the study of natural phenomena.

Currently, the study of complex systems (physical, biological and psychosocial systems) transcends the mechanistic and reductionist methods for describing linear processes and requires suitable approaches to describe probabilistic and scarcely predictable phenomena [1,11,12,13,14,15,16,17,18,19,20].

The term theory of complexity was used for the first time in an article published in 1978 in *Scientific American* [21]. This work showed that all systems characterised by numerous variables that interact with each other according to some form of internal organisation are non-linear, unpredictable and probabilistic and show common features regardless of their nature.

Since the 1970s, statistical mechanics, chaos theory and general systems theory have laid the foundations for a necessary methodological revision. Since the beginning of the third millennium, several other research methods have become the focus of scholars: the theory of complex networks [22,23,24], neural networks for deep learning [25,26,27,28], decision trees [29,30,31] and cluster analysis methods [32,33,34].

These approaches to complexity are predominantly algorithmic and can be poorly formalised in mathematical terms. This highlights an isomorphism between natural phenomena and the methods used to describe them. It has become evident that iterations and computational flows of these methods cannot be crystallised in formulas in the same way that it is not possible to crystallise in precise definitions the relationships and interactions between the parts of complex systems.

These methods were born with the aim of simulating the activity of neuronal networks and have shown great utility as descriptive means of natural phenomena. At the same time, they have shown themselves capable of emulating natural processes and have given a great and definitive impulse to the field of artificial intelligence research [35,36] and are opening the way to research fields such as computational psychology [37,38] and more recently, computational psychotherapy [39,40,41].

The scientific value of these methods can be found precisely in the renunciation of the claim to create precise formal models applicable to facts of nature, being content with an acceptable approximation in the description of stochastic phenomena. Paradoxically, from this acceptance of uncertainty emerged the ability of networks to describe and predict the evolution of systems.

### 1.2. Aim

In this review, we focus on the recent literature on computational methods in psychotherapy. This work reviews psychotherapy process studies that used computational methods to analyse their data. Starting from the assumption that linear methods are not adequate for the study of complex phenomena such as those occurring in the psychotherapeutic process, our aim is to gather new developments in the field of computational methods in psychotherapy. We believe that an integrated set of results on recent developments about this topic offers the possibility of a better understanding of the phenomena occurring in psychotherapeutic interactions as well as in human interaction more generally. In doing so, we also aim to form a basis for improving psychotherapy research and practice by providing some tentative recommendations for further evaluation.

## 2. Methods

### 2.1. Search and Retrieval

Scoping reviews are to be understood as an increasingly popular means of synthesising the existing literature on a topic or field where there is a lack of rigorous evidence, with the aim of quickly mapping the key concepts underpinning that area of research. Scoping reviews are an optimal tool for identifying the existence of a sample of literature on a given topic and providing an overview (broad or detailed) of its focus; they are useful for examining emerging evidence when it is not yet clear what more specific questions can be asked and addressed by a systematic review.

We conducted a scoping literature review following PRISMA Extension for Scoping Reviews (PRISMA-ScR) guidelines [42], and this model comes to life through 20 criteria (plus two optional ones) that the researcher is asked to answer in order to have his or her work identified within the Scoping Reviews category.

The aim was to retrieve all articles published in English peer-reviewed journals related to interpersonal coordination occurring in computational psychotherapeutic methods that were published during the period between January 2011 and January 2022. The choice of this precise time frame is due to having noticed that most of the articles on this topic have been published since 2011. We have chosen our search terms on the basis of previous research and on articles deemed a priori relevant.

The search was carried out from 15 January 2022 to 15 February 2022. Sources coming from the different repositories were merged in a single dataset after removing duplicates.

The searches were conducted separately on the basis of the following terms: computational methods in psychotherapy. The Boolean operator used was “and”. Searches based on these keywords were conducted in PubMed and Scholar. 

The eligibility and exclusion criteria are listed below (Table 1). Limitations of the review, by definition, are that articles outside of our search terms, time period or eligibility criteria were not included.

### 2.2. Study Selection

The search mechanism of the results was imputed in Rayyan [43] and selected according to the inclusion criteria in two stages. In the beginning, studies were selected or excluded based on the titles and abstracts of each entry. Studies with insufficient information proceeded to stage two. 

In the second phase, these designated studies were finally selected based on reading the full text.

The selection was carried out by two independent researchers, and conflicts were resolved through discussion between the two referees or, in the case that they could not reach a firm decision, by the assessment of a third researcher, selected on the basis of his or her expertise in the topic of the question.

### 2.3. Assessment of Methodological Quality 

According to the PRISMA guidelines for scoping review, the assessment of the methodological quality of the studies was not applied.

### 2.4. Data Extraction and Selection

The execution of this review had the support of 4 experts, one in psychiatry and in the modelling of complex systems, two in psychotherapy process research and one in psychology, who specialised in machine learning.

The selected studies were imputed into a new Rayyan project for data extraction. During the data extraction procedure, the full text of the papers was read and we used the data extraction form to extract the features of interest.

Again, the selection was performed by two independent researchers, and conflicts were resolved by discussion between these two researchers or, when they could not reach a firm decision, by the intervention of a third researcher, who was always selected based on his or her expertise on the topic. Since at the end all the decisions were shared on a collegial level, it was not necessary to perform the inter-judge reliability.

The screening process had a first stage in which the researchers read only the title and the abstracts (title and abstract screening) and a second stage in which the researchers read also the full text (full-text screening). 

Of the 893 results obtained during the abstract screening phase, only 87 seemed to investigate the use of computational methods in psychotherapy, and thus were eligible and accessed in full text. Among these 87 papers, 57 were excluded in the first phase, based on the previously described exclusion criteria. A further selection was used to exclude a further 12 articles, which were mainly theoretical (without data), except for one which was in German and was therefore excluded as a non-English publication, according to the eligibility criteria. Finally, it was possible to identify 18 publications that were included in our scoping review. 

### 2.5. Data Charting Process

The results are presented through tables, graphs, diagrams and narrative descriptions. To reorder the data, it was very useful to start with the representation in a table with which we summarised the results schematically. In general, the purpose of the other tables, graphs and diagrams was to summarise the relevant data obtained, providing an overview of the various methods used.

### 2.6. Critical Appraisal of Individual Sources of Evidence

The sources of evidence, before being included, were critically evaluated, in particular those sources were chosen that were actually written in an objective manner [44], i.e., able to highlight in a transparent and verifiable manner methods and results of research on computational methods in psychotherapy, according to the universally recognised criteria of the scientific community, and subsequently validated according to the rules of peer review.

## 3. Results 

### 3.1. Selection of Sources of Evidence 

**Figure 1:**
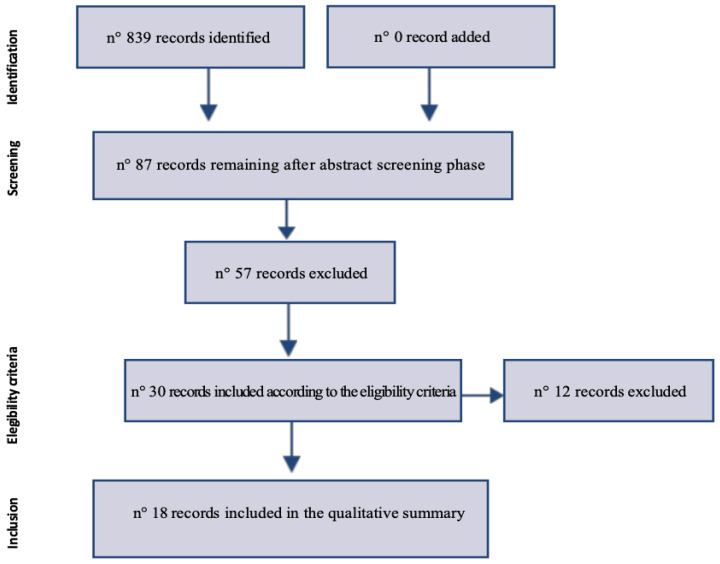
Selection of sources of evidence flow chart (n°: means “numbers”).

A total of 18 sources of evidence were screened, assessed for admissibility and finally included in the review. At the first stage we excluded only those documents whose abstract showed that they were not relevant to the topic, books and book chapters. Subsequently, we also excluded opinion papers, literature reviews, research that did not really include the use of computational methods in psychotherapy, research where the intervention was carried out through virtual agents that do not have a human subject, papers whose data analysis was not suitable for the scoping review process and articles that, despite having an English abstract, did not have the text written in English. This process is graphically described in the flow chart (Figure 1).

### 3.2. Characteristics of Sources of Evidence 

We grouped the studies according to the types of computational methods used. We divided the articles into two types: (a) articles that used computational tools for psychotherapy; (b) articles that used statistical computational methods (computational techniques) to analyse processes that occurred in psychotherapeutic settings. We have summarised the type of settings, study aim, sample, data analysed and conclusions, as shown below (Table 2).

### 3.3. Results of Individual Sources of Evidence

The 18 selected papers can be divided into three groups. 

In particular, we have:

I. A first group of four papers that used only computational experimental approaches with the use of digital tools or machine learning;
II. A second group of six papers that used complex statistical methods;
III. A third group of eight papers that used combined computational and experimental approaches, i.e., both complex statistical methods and computational devices. All but one of the studies collected measured data.

*I group.* Of the four papers that used only computational experimental approaches, with the use of digital tools or machine learning, three explored the characteristics of online psychotherapy, evaluating both patient and therapist perspectives [45,46,47], and in all three the feasibility of providing regular psychological assistance with the necessary adaptations to the patient’s circumstances using an IT device was highlighted. In contrast, the fourth paper focused on attempting to personalise the treatment of depression by developing specific computational models that use electronic health record (EHR) data to predict the diagnosis and severity of depression and response to treatment [48]. The authors pointed out that it is possible to use EHR data to predict a diagnosis of depression up to 12 months in advance and to differentiate between extreme levels of depression. 

*II group.* Of the six articles that used statistics relating to the theory of complex systems for data analysis, there was one that [41], applying a mathematical model that combined five variables essential in the psychotherapeutic process (intensity of emotions, intensity of the problem, motivation to change, insight and new perspectives, therapeutic success) and four parameters in a set of five coupled non-linear differential equations. This model resulted in chaotic dynamics, phase transition phenomena, bi- or multi-stability and sensitivity of the dynamic models to parameter drift. All characteristics belonging to chaos theory. The authors concluded that therapeutic processes are complex phenomena that require technologies capable of monitoring and reporting in real time on non-linear features of the ongoing process (e.g., its stability or instability). Three of these last six papers tried to evaluate the possibility of describing the complexity of therapeutic relationships using the methods of machine learning and complex networks (GEPHI networks; BERT, bidirectional encoder representations from transformers; topic model) [39,49,50]. All three computational methods used showed a high level of accuracy in analysing psychotherapeutic sessions. Cella et al. [51] identified corrective cognitive change through computational modelling, exploring the effects on reinforcement learning in schizophrenia. This study was a cross-sectional design comparing scores obtained on the Wisconsin Card Sorting Test and computational modelling parameters in a group of individuals with schizophrenia with a group of healthy controls. Model parameters were estimated by maximum likelihood estimation using previously published methods. The authors found that schizophrenia reinforcement learning difficulties negatively influenced performance on turn-based learning tasks in patients with schizophrenia and that cognitive remediation therapy (CRT) could instead improve sensitivity to reward and punishment. The identification of the parameters showing change following psychotherapeutic intervention constituted evidence that could be used in experimental medicine studies to identify cognitive domains susceptible to improvement in patients with schizophrenia and thus target them in a more focused manner. Portêlo et al. [52] attempted to mathematically characterise changes in fear ratings in patients (30 women with spider phobia) who underwent virtual exposure therapy sessions, and then sought to understand whether the resulting model would help predict treatment outcome. They selected the best model using Bayesian techniques. The best model also made remarkably accurate predictions for the test exposure, while one of the model’s parameters helped predict the treatment outcome. These authors concluded that individual patterns of fear change during exposure therapy and can be characterised mathematically, stating that this mathematical characterisation helps predict the treatment outcome.

*III group.* The remaining eight papers combined the use of complex statistical methods with experimental designs using computational devices. Of these last ones, three papers [53,54,55] used a combination of devices based on sensorimotor and sensorineural feedback to guide different learning tasks, developing interactive multimodal models. Subsequently, the researchers identified algorithms capable of understanding how human neurosensorimotor functioning is coupled to multiple and simultaneous modes of feedback modalities. The experiments were based on the construction of interactive environments specifically designed to guide learning. These studies attempted to build a computational framework for the application of virtual information to assist motor learning for complex tasks requiring the coupling of different perceptual experiences, in an attempt to develop a computational tool to assist neurorehabilitation therapy and/or psychotherapy in order to generate effective and personalised rehabilitation and treatment strategies. The studies by Reiter et al. [56] and Frässle et al. [57] were also conducted using integrated protocols; however, the data analyses were performed using Bayesian model selection. Price et al. [55] applied a well-validated form of computational modelling (drift-diffusion model; DDM) to trial-level reaction time data from a two-choice dot-probe task—the dominant paradigm used in hundreds of studies of attention—in order to model the distinct components of task performance, in a sample of anxious subjects. This new analytical approach of theirs resulted in much improved split-half reliability, modestly improved test-retest reliability and revealed new mechanistic insights concerning the neural substrates of attentional bias and the impact of an attentional retraining procedure. The authors concluded that the computational modelling of attentional bias task data may represent a new avenue for improving the accuracy of component modelling. Similarly, Berardi et al. [58] developed a computational behavioural model to aid the formalisation and automation of behavioural modelling procedures within adaptive behavioural interventions using real-time technology. Digital experiments were performed with this model for a range of parameters in order to identify features that optimally generated the behaviour. Finally, the group of Tschacher et al. [59] also used a computational model combined with an experimental biofeedback approach. The psychophysiological synchrony between patient and psychotherapist during psychotherapy sessions was measured. Synchrony analyses were conducted using two methodological approaches, the calculation of cross-correlations (surrogate synchrony [SUSY]) and slopes (surrogate concordance [SUCO]). The results support the existence of a physiological synchrony in this collection of psychotherapy sessions, which speaks to the sympathetic and parasympathetic coupling between this therapist and her clients, and its link to assessments of the therapeutic process. Moreover, the feasibility of deriving synchrony signatures of physiological signals with the described methodology was confirmed.

### 3.4. Synthesis of Results 

The summary of the results of the review is displayed in Table 3.

## 4. Discussion

Drawing on recent literature on the potential of computational approaches in psychotherapy, this review represents an attempt to systematise the empirical literature on applications of computational methods in psychotherapy research. Specifically, our intent was: (1) to provide clinicians with information on the methods and applications of different computational methods in the context of psychotherapy; (2) to shed light on the usefulness of these methods and the possibilities of their use in the various fields of application of psychotherapy research; and (3) to highlight the clinical implications and then attempt to identify potential opportunities for further research.

Eighteen studies were identified in a systematic search through journals specialising in mental health. Classification of the studies on the basis of the experimental protocol and complex statistical methods used was not possible due to the heterogeneity of the studies.

However, it was very interesting to discover that, regardless of the computational model used, all the studies confirmed the usefulness of predictive and learning models in the study of complex variables such as those belonging to psychological, psychopathological and psychotherapeutic processes. The latter evidence can be found in Table 3 in the conclusions column. Particularly, protocols that had combined the use of complex statistical methods with biofeedback experimental designs, using computational devices, were of great applicative interest for research in psychotherapy.

In particular, these studies show that using machine learning for the development of interactive multimodal models can direct future research in order to personalise treatments and a more accurate planning of the treatment plan that includes changes during the psychotherapy path, thanks to the identification of process markers capable of predicting outcomes [35,39,40].

Most of the studies identified in this scoping review should be considered the first works demonstrating the potential use of computational methods to address questions about psychotherapy and what types of algorithms get the best performance. Caution is needed to avoid over-interpretation of preliminary results. The use of computational methods, taken alone, does not necessarily increase the chances of success of the treatment or improve clinical decision making, but collects diagnoses information, clarifying the processing processes. By examining the therapist’s behaviour, computational methods can have an impact on traditional approaches to the provision of psychotherapy [61]. Compared to traditional statistics methods, computational methods offer new possibilities for analysis of big data and better and more advanced computing performance, so that the advantages of computational methods probably outweigh the errors that inevitably occur in classical statistical models [43,62].

In addition, evidence that comes from neurorehabilitation that uses biofeedback can give important suggestions about how can use biofeedback in the research about the phenomenology of the psychotherapeutic meeting [61]. In fact, some research, which is studying, for example, the characteristics of empathy in both distance and presence-based psychotherapy, has considered it a desirable frontier to be able to measure the psychophysiological variables of the patient and therapist in order to verify their attunement [47]. Other studies, starting from the concept that the observation of movement in human beings (such as gestures and actions performed in the relational context) allows us to detect the most significant and evident expression of the complexity of a relationship, have also offered interesting insights by exploring the motor aspects of certain dyads [63]. For this reason, it was deemed interesting to include some studies that reconstructed a computational framework for constructing interactive feedback for assisting motor learning, as they could give interesting insights to study and analyse those kinds of psychotherapeutic interactions based on phenomenological leaning or who base their interventions on the philosophical principles of enactivism and the embodied mind [64].

### Limitations

The limitations encountered in the process concerned the difficulty in standardising the results of the review with reference to some parameters. In particular, the selected articles differed with respect to the methods of processing experimental data, the topics, the sampling, the tools and the experimental protocols used. The reason for these differences is to be found in the novelty of the topic and in the consequent need for time for experimentation.

## 5. Conclusions

From this scoping review it is possible to conclude that there is still no unambiguous direction regarding the use of computational methods in psychotherapy, and that there are many and varied methods used by various authors that are difficult to summarise. However, while this evidence tells us that there are numerous opportunities in the field, at the same time more evidence and shared guidelines are needed to disseminate shared and reproducible protocols.

For this reason, it is possible to affirm that further clinical research collaborations are needed to develop specific algorithms for different treatments and patient groups. Moreover, further opportunities need to be identified for the applications of computational methods to improve the progression of processes and results in psychotherapy. Of course, there remains a need to consider ethics regarding the collection, analysis and sharing of processing data, as well as the implementation of feedback tools based on computational methods and machine learning in clinical practice.

In general, it can be stated that complex statistics algorithms and protocols need to be continually refined and improved, but certainly they could provide help to therapists in the early diagnosis of some mental illnesses, to build personalised and early treatment plans based on the peculiar characteristics of an individual.

Finally, the most important evidence would be to enable therapists to focus on the relational aspects of psychotherapy, considering that complex encounter between patient and therapist, which can only be achieved through the reciprocity of therapist–patient interactions.

## Figures and Tables

**Table 1:** Eligibility and Exclusion Criteria.

Eligibility Criteria	Exclusion Criteria
1. Published in English	1. Books
2. Published in a peer-reviewed journal	2. Editorial
3. Empirical examination of dyadic psychotherapy sessions or interviews between patient (or pseudo-patients based on actual patients) and therapist	3. Opinion papers
4. Quantitatively assessed temporal relationships in measures collected from patient and therapist	4. Literature reviews
5. Empirical research of the use of computational tools for the psychotherapy sessions	5. The research did not really include the use of computational methods in psychotherapy
	6. The intervention was carried out through virtual agents that do not have a human subject
	7. Data analysis was not suitable for the scoping review process

**Table 2:** Prototype table.

Title	Year	First Author	Study Aim	Sample	Data Analysed	Computational Technique	Computational Methods and Tools/Devices	Conclusions

**Table 3:** Synthesis of results.

Title	Year	First Author	Study Aim	Sample	Data Analysed	Computational Technique	Computational Methods and Tools/Devices	Conclusions
A computational framework for constructing interactive feedback for assisting motor learning [53]	2011	Hari Sundaram	To identify and characterise different sensorimotor control strategies used by normal individuals and by hemiparetic stroke survivors acquiring a skilled motor task.	Normal individuals and hemiparetic stroke survivors	Novel interactive tasks environment in which subjects are provided with rich auditory and visual feedback of movement variables to drive motor learning	SLEP package The algorithms in SLEP achieve the optimal convergence rate among all first-order methods and scale to high-dimensional data.	Sparse inverse covariance estimation. A linear regression model estimates the interaction between a specific variable and the remaining variables.	The resulting computational framework will have significant impact on advancing smart neurorehabilitation. The frame-work will allow computer-assisted, continuous customisation of therapy based on the best available evidence
Action and object words are differentially anchored in the sensory motor system A perspective on cognitive embodiment [54]	2018	Houpand Horoufchin	To use the MVPA and the predictive pattern decomposition analysis techniques to find out whether a common neural activation pattern exists in the human brain for nouns and objects, or verbs and actions, respectively.	Twenty healthy native German speaking participants (10 female; mean age 24.4 years; SD 3.14; range, 18–31 years)	Participants were exposed to: Pictorial stimuli depicting (1) plain objects and (2) objects with hand–object interactions, and the corresponding words, (3) nouns and (4) verbs. A linear classifier was trained on half the 4800 neural activity maps, thus 2400 examples of training data, neural activity maps from 1200 experimental trials with plain textual stimuli with verbs versus nouns.	A toolbox of data-driven machine learning techniques that allowed the automatic extraction of useful neural patterns from fMRI recordings. Functional MRI data were acquired from healthy participants. The data were analysed using RFE, applied on the data using a mask obtained from an ALE meta-analysis	SVM Mean averaging across prediction instances yielded out-of-sample performance and binomial-tested *p*-values. All statistical-learning analyses were performed in Python. Scikit-learn provided efficient, unit-tested implementations of state-of-the-art statistical learning algorithms.	The results support the hypothesis that, functionally, mirror neurons and canonical neurons act in parallel and in very close anatomical proximity. Further, these results confirm the predictions of embodied and grounded cognition theories. Based on neural recycling theories, which are long embraced by the experimental psychology communities, the results demonstrate that words, such as verbs and nouns, are grounded in the sensorimotor system, and that they activate the canonical and mirror neuron systems in subtly different ways.
Computational Modeling Applied to the Dot-Probe Task Yields Improved Reliability and Mechanistic Insights [55]	2018	Rebecca B. Price	DDM could produce a purer behavioural measure of the attentional patterns of interest, and might yield more precise and/or psychometrically sound estimates, enabling potential reanalysis of response data from many hundreds of studies that have previously utilised the dot-probe task in the study of psychopathology.	Seventy unmedicated patients reporting clinically elevated levels of trait anxiety and associated clinician-rated disability were randomised to receive active ABM (n = 49) or a sham control variant (n = 21).	Two conditions of shorter (500 ms) duration trials, comprised of 60 trials each, which were randomly interspersed over the course of the experiment	Analyses were completed using *fast-dm* software.	A well-validated form of computational modelling DDM. To provide a direct measure of overt eye movements, a RK-768 eye-tracker concurrently measured eye gaze during the task. In a separate fMRI session prior to the onset of treatment, the same dot-probe task was administered, with minor modifications.	While DDM-derived attentional bias indices exhibited convergent validity with previous approaches, this novel analytic approach yielded substantially improved split-half reliability, modestly improved test–retest reliability and revealed novel mechanistic insights regarding neural substrates of attentional bias and the impact of an automated attention retraining procedure. Computational modelling of attentional bias task data may represent a new way forward to improve precision.
Computational model for behaviour shaping as an adaptive health intervention strategy [58]	2018	Vincent Berardi	To develop computational models that are suitable for a JITAI framework. This is accomplished by modifying McDowell’s evolutionary model of behaviour dynamics by incorporating behaviour shaping.		To operationalise the construct of behaviour shaping within the modified version of the McDowell computational model so that digital experiments concerning its optimal implementation can be performed. To simulate the reinforcing of successive approximations to a target behaviour.	Continuous shaping procedure: modifying McDowell’s evolutionary model of behaviour dynamics by incorporating behaviour shaping.	Digital experiments were performed with this updated model for a range of parameters in order to identify the behaviour-shaping features that optimally generated target behaviour.	This work demonstrated the viability of using computational models to investigate behaviour-shaping routines. The results indicate that shaping was more effective at engendering higher levels of target behaviour than when only the target behaviour class was reinforced.
Physiological synchrony in psychotherapy sessions [59]	2020	Wolfgang Tschacher	To explore physiological synchrony in naturalistic psychotherapy sessions and the association of such synchrony with self-report ratings.	55 dyadic psychotherapy sessions conducted by one	Entire sessions (average duration, 51 min) were assessed for physiological synchrony of therapist’s and client’s respiration, electrocardiogram, heart rate and heart rate variability.	Synchrony analyses were conducted using two methodological approaches, computation of cross-correlations SUSY and of window-wise slopes SUCO.	Two methods of synchrony computation were applied to the time series: windowed cross-correlation and correlation of local slopes (concordance). Both methods included surrogate controls using segment-wise shuffling.	Results support the existence of physiological synchrony in this collection of psychotherapy sessions, which speaks for the sympathetic and parasympathetic coupling between this therapist and her clients and its link with ratings of the therapy process. The feasibility of deriving signatures of synchrony of physiological signals with the described methodology was corroborated.
Decoding attentional states for neurofeedback: mindfulness vs. wandering thoughts [60]	2018	Zhigalov A	The authors used MEG to acquire brain activity during mindfulness meditation and thought-inducing tasks mimicking wandering thoughts and novel real-time feature extraction to decode the mindfulness to discriminate it from the thought-inducing tasks. To investigate whether and how it is possible to discriminate (decode) between MF, FP.	Twenty-four subjects with no history of neurological disorders, head trauma or substance abuse	Subjects’ responses for the question “How focussed were you on the task?” ranged from 0 to 1. The average values were the following: 0.65 ± 0.012 (mindfulness), 0.70 ± 0.010 (future planning task) and 0.66 ± 0.013 (anxiety-inducing task), respectively.	Complex-valued independent component analysis in the frequency-domain Fourier-ICA, and an algorithm described by Kauppi and colleagues	Planar gradiometers of the MEG scanner. The SSS method was applied to suppress the external interference and sensor artefacts.	The authors developed spectral- and connectivity-based classification approaches and showed that the mental states underlying mindfulness and thought-provoking tasks can be discriminated using MEG recordings and machine learning approaches.
Impaired Flexible Reward-Based Decision-Making in Binge Eating Disorder: Evidence from Computational Modeling and Functional Neuroimaging [53]	2016	Andrea M.F. Reiter	Performing fMRI analysis informed via computational modelling of choice behaviour, the authors identified specific signatures of altered decision-making in BED.	Twenty-two BED patients and 22 healthy control subjects	During fMRI, participants performed 160 trials of a decision-making task designed to examine flexible behavioural adaptation.	The tested model space included four variations of RL-models. These models update expectations via prediction errors (PEs), which quantify the mismatch between actual outcome and prediction. Model-free PEs are only computed for chosen stimuli, although PEs can also be computed for the unchosen stimulus.	Data analyses were performed using MATLAB R2012, IBM SPSS Statistics for Windows, Version 22 and R 3.2.0. Bayesian model selection.	The results, which combined fMRI and computational modelling of reinforcement learning, provide novel insight into the neural correlates of maladaptive decision-making in BED, thereby helping to refine a neurocognitive phenotype of the newly classified disorder. They observed impaired behavioural adaptation in a dynamic environment in BED as compared to healthy controls. By adopting a computational psychiatry approach combined with modelling-informed fMRI analysis, this study contributes to refining the neurocognitive phenotype of BED as an addition to clinical observations and new diagnostic criteria in the *DSM-5*.
Test-Retest Reliability of Effective Connectivity in the Face Perception Network [57]	2016	Stefan Frässle	A thorough investigation of the test–retest reliability of an fMRI paradigm for DCM analysis dedicated to unravelling intra- and interhemispheric integration among the core regions of the face perception network.	25 healthy volunteers	The reliability of face-specific BOLD activity in volunteers, performed with a face perception paradigm, was examined. They assessed the stability of effective connectivity among specific regions by analysing the reliability of Bayesian model selection and model parameter estimation in DCM	DCM is a Bayesian framework for investigating the effective connectivity within a neural net- work based on neuroimaging data.	Using the Presentation 11.0 software, all stimuli were presented as circular patches (diameter: 4.34 deg) on an MRI-compatible LCD screen (LG SL9000, 60 Hz, 4:3, 10,243,786 pix). Imaging data were acquired on a 3-Tesla MR scanner. Analyses of functional imaging data were conducted using SPM8. The statistical analysis of BOLD activations was conducted using a first-level GLM.	The fMRI paradigm presented provides a reliable approach for investigating effective connectivity in the core face perception network by taking into account the intra- and interhemispheric integration among the core regions. This approach might therefore prove valuable for understanding face processing at the individual-subject level.
Satisfaction degree in the using of VideoConferencing Psychotherapy in a sample of Italian psychotherapists during COVID-19 emergency [46]	2020	Cioffi Valeria	To analyse the degree of satisfaction after using VCP in a sample of psychotherapists freely recruited through the publication on social media of a specially created questionnaire.	507 psychotherapists	This study, analysing the responses to a satisfaction questionnaire published online by psychotherapists from all over the Italian territory, identified specific characteristics of psychotherapists able to predict their degree of satisfaction in using VCP during the COVID-19 pandemic.	CRT	VCP	The first two important characteristics of the psychotherapists to influence the satisfaction in their use of VCP (belonging to the specific age group 45–65 and having previously used VCP) are probably a matter that can be linked to the degree of professional maturity and experience. To have a certain level of proficiency, according to their own orientation, in the use of the VCP may influence the level of satisfaction of the psychotherapists.
Exploring the Question: “Does Empathy Work in the Same Way in Online and In-Person Therapeutic Settings?” [47]	2021	Sperandeo Raffaele	To analyse the degree of empathy between the psychotherapist and client pair, and the degree of support perceived by the client who was referred to as the patient interchangeably in this study, comparing the sessions in person with those online, during the current pandemic, in order to discriminate the impact of empathy in the digital setting.	23 patients with different severity of pathology, engaged in online and in-person therapeutic sessions, with five psychotherapists of different theoretical leanings	The perception of empathy and support was evaluated in parallel in the two members of the 24 therapeutic couples after four consecutive sessions. Empathy and support perceived by patient and therapist were assessed after 72 therapy sessions (39 remotely; 33 in person).		VCP	Unlike the psychotherapists, the patients perceived their therapists as significantly more empathic and supportive in the remote setting. These are rather important data, because the literature documents that client empathic perception measures represent a more accurate measure of the empathic relationship and, in general, can predict a good treatment outcome.
Toward personalizing treatment for depression: predicting diagnosis and severity [48]	2014	Huang S.H.	To develop and evaluate computational models that EHR data for predicting the diagnosis and severity of depression, and response to treatment.	Two datasets: 35,000 patients (5000 depressed) from the Palo Alto Medical Foundation and 5651 patients treated for depression from the Group Health Research Institute.	A develop regression-based models for predicting depression, its severity and response to treatment from EHR data, using structured diagnosis and medication codes as well as free-text clinical reports.	LASSO Logistic regression from the R glmnet package.	HER R glmnet package.	It is possible to use EHR data to predict a diagnosis of depression up to 12 months in advance and to differentiate between extreme baseline levels of depression.
Case Report Of A Computer-Assisted Psychotherapy Of A Patient With Als [45]	2014	Ana Isabel García Pérez	This case describes a psychotherapy intervention in a patient in advanced stages of ALS. The inability for verbal communication at these stages necessitated the inclusion of a computational system to favour AAC to provide psychological care.	One 66-year-old woman diagnosed with ALS	The computer-assisted language device was used to deal with the complex topics that arise in psychotherapy in the advanced stage of the disease.		Boardmaker with Speaking Dynamically Pro Bimodal approach including an AAC device in association with psychotherapy	The novelty of this communication is to report how the regular psychological care could be adapted to the patient’s circumstances using a computer device. Psychotherapy protocols using AAC need to be evaluated in cohort or clinical studies in order to determine their suitability for the majority of patients with ALS. The present case report intends to be a contribution to this field.
An Automated Quality Evaluation Framework Of Psychotherapy Conversations With Local Quality Estimates [50]	2021	Zhuohao Chen	To propose a hierarchical framework to automatically evaluate the quality of a CBT interaction.	1118 CBT sessions 4268 UCC sessions	Sessions were split into blocks, and BERT was employed to learn segment embeddings, and use those features within a LSTM-based model to make predictions about session quality.	BERT		The experimental results suggest that incorporating the local quality estimator leads to better segment representations and to consistent improvements for assessing the overall session quality in terms of most of the CTRS codes. The authors discuss how the estimated scores of the segment benefit the prediction tasks by comparing the differences of the segments within the same session.
The nodes of treatment. A pilot study of the patient-therapist relationship through the theory of complex systems [49]	2021	Raffaele Sperandeo	To evaluate the possibility of describing the complexity of therapeutic relationships using the methods of machine learning and complex networks	28 psychotherapy sessions of seven psychotherapist–patient couples	The nodes were identified thanks to the subscales of a phenomenological self-observa- tion test, defined as the ACL. The tool consists of a list of 300 adjectives, from which the subjects can select those they consider to be referable to their person. The graphs produced by each session were analysed and compared with each other.	The networks were designed using GEPHI. The connections between the nodes were evaluated by measuring the Euclidean distance between the subscale scores. Non-oriented graphs were constructed with uniquely positive and reciprocal connections.		The use of graphs is a valid tool for the analysis of both the psychotherapeutic sessions and the evolution of the care relationship over time. Numerous suggestions on the dynamics within the patient–therapist system emerge from the construction of a complex network useful for describing the trend of psychotherapy, which in this way can be described without losing the value of the wealth of each individual experience.
Computational Psychotherapy Research: Scaling up the Evaluation of Patient–Provider Interactions [39]	2015	Zac E. Imel	To verify that: topic models would estimate clinically relevant semantic content in therapy transcripts; if semisu-pervised models could identify semantically distinctive content from different treatment approaches; to classify treatment types of new psychotherapy sessions automatically.	Transcripts from 1553 psychotherapy and psychiatric medication management sessions	Transcripts from 1553 psychotherapy and psychiatric medication management sessions	To evaluate the potential of topic models to “learn” the language of psychotherapy, two different types of topic models were applied.	Topic Models	The topic model classified treatment sessions with a high degree of accuracy.
Identifying Cognitive Remediation Change Through Computational Modelling—Effects on Reinforcement Learning in Schizophrenia [51]	2014	Matteo Cella	Converging research suggests that individuals with schizophrenia show a marked impairment in reinforcement learning, particularly in tasks requiring flexibility and adaptation. The problem has been associated with dopamine reward systems. This study explores, for the first time, the characteristics of this impairment and how it is affected by a behavioural intervention—cognitive remediation.	Patients with a diagnosis of schizophrenia (*N* = 100)	This study is a cross-sectional design comparing WCST scores and computational modelling parameters in a group of individuals with schizophrenia to a group of healthy controls.	Model parameters were estimated through *Maximum Likelihood Estimation* using previously published methods.		Schizophrenia reinforcement learning difficulties negatively influence performance in shift learning tasks. CRT can improve sensitivity to reward and punishment. Identifying parameters that show change may be useful in experimental medicine studies to identify cognitive domains susceptible to improvement.
Mathematical Characterization of Changes in Fear During Exposure Therapy [52]	2021	Ana Portêlo	During exposure therapy, patients report increases in fear that generally decrease within and across exposure sessions. The main aim was to characterise these changes in fear ratings mathematically; a secondary aim was to test whether the resulting model would help to predict treatment outcome.	30 treatment women with spider phobia and no comorbidities.	Patients were randomly assigned to one of two groups: single context (n = 15), in which the spider was presented in the same room colour in all training exposures, or multiple contexts (n = 15), in which the room colour was different on each training exposure. Patients completed the German version of the FSQ before and after treatment.	A hybrid data- and theory-driven approach was used by using data to select the best among a set of theoretical models.	First investigated the component processes using classical statistics. The classical statistical analyses showed no evidence for a change in return of fear across exposure intervals during the training period. Then used the VBA toolbox for Bayesian model selection. Linear regression to test whether model parameters obtained for individual patients could predict treatment outcome, defined as the change in FSQ score from pre- to post treatment.	Computational psychiatry encompasses data- and theory- driven approaches, which can be combined. In the model, fear within exposures decreases following a differential equation in which the instantaneous fear decrease rate is proportional to the fear level.
Psychotherapy Is Chaotic—(Not Only) in a Computational World [41]	2017	Günter K. Schiepek	The aim of this article is to outline the role of chaotic dynamics in psychotherapy.	Common factors of psychotherapeutic change and psychological hypotheses on motivation, emotion regulation and information processing of the client’s functioning can be integrated into a comprehensive non-linear model of human change processes.	The model combines five variables (intensity of emotions, problem intensity, motivation to change, insight and new perspectives, therapeutic success) and four parameters into a set of five coupled non-linear difference equations. The results of these simulations are presented as time series, as phase space embedding of these time series (i.e., attractors) and as bifurcation diagrams.	The system was programmed in Excel 2007 and for reasons of validation also in Matlab.	The model covers 16 functions connecting five variables. The functions are represented in mathematical terms, which are integrated into five coupled non-linear difference equations. Each equation describes the development of a variable, depending on other variables, on itself and on the involved parameters.	The model contributes to the development of an integrative conceptualisation of psychotherapy. It is consistent with the state of scientific knowledge of common factors, as well as other psychological topics, such as: motivation, emotion regulation and cognitive processing. The role of chaos theory is underpinned, not only in the world of computer simulations, but also in practice. In practice, chaos demands technologies capable of real-time monitoring and reporting on the non-linear features of the ongoing process (e.g., its stability or instability).

**Legend:** SLEP: sparse learning with efficient projections; MVPA: multivariate pattern analysis and confounding in neuroimaging; RFE: recursive feature extraction; SVM: trained binary classification algorithm; DDM: drift-diffusion model; JITAIs: just-in-time adaptive interventions; SUSY: surrogate synchrony; SUCO: surrogate concordance; MEG: magnetoencephalography; SSS: signal space separation; MF: mindfulness meditation; FP: future planning; BED: binge eating disorder; DCM: dynamic causal modelling;  SPM8, version 3042: statistical parametric mapping; GLM: general linear model; CRT: classification regression trees; VCP: videoconferencing psychotherapy; LASSO: least absolute shrinkage and selection operator; EHR: electronic health record; ALS: amyotrophic lateral sclerosis; AAC: augmentative and alternative communication; BERT: bidirectional encoder representations from transformers; ACL: adjective checklist; FSQ: Fear of Spiders Questionnaire; VBA: variational Bayesian analysis.

## Data Availability

The searches were conducted separately on the basis of the following terms: computational methods in psychotherapy. The Boolean operator used was “and”. Searches based on these keywords were conducted in PubMed (https://pubmed.ncbi.nlm.nih.gov/) and Scholar (https://scholar.google.com/).
